# Effect of Acute Surgical Stress on Serum Ghrelin Levels

**DOI:** 10.4021/gr455e

**Published:** 2012-05-20

**Authors:** Nikolaos Kontoravdis, George Vassilikostas, Emmanuel Lagoudianakis, Apostolos Pappas, Charalampos Seretis, Nikolaos Panagiotopoulos, Nikolaos Koronakis, John Chrysikos, George Karanikas, Ioannis Manouras, Ioanis Legakis, Dionysios Voros

**Affiliations:** a Second Department of Surgery, Aretaieion University Hospital, University of Athens, Greece; b Second Department of Surgery, 401 Army General Hospital, Athens, Greece; c 1st Department of Propaedeutic Surgery, Hippokrateion Hospital, Athens Medical School, University of Athens, Q. Sofias 114 avenue, 11527 Athens, Greece; d Second Department of Surgery, 417 NIMTS (Military Veterans' Fund Hospital), Athens, Greece; e Department of Endocrinology, Henry Dunant Hospital, Athens, Greece

**Keywords:** Ghrelin, Stress, Abdominal surgery, Cholecystectomy, Colectomy

## Abstract

**Background:**

Ghrelin is an appetite hormone that influences the gastrointestinal function and regulates energy metabolism. Growing evidence also suggests that this hormone plays a central role in immune modulation. Each surgical operation is followed by a series of inflammatory and metabolic changes that constitute the stress response. The aim of our study is to evaluate the effect of stress during different types of abdominal surgery in ghrelin serum levels.

**Methods:**

An overall of 25 patients were prospectively allocated in two groups based on the type of surgical operation. Group A (n = 10) patients were scheduled to undergo cholecystectomy, whereas Group B (n = 15) patients underwent colectomy. Serum ghrelin concentrations were evaluated in each patient preoperatively, after the induction of general anesthesia and tracheal intubation, one and five hours after the beginning of surgery and the morning of the first and second postoperative day.

**Results:**

In both groups serum ghrelin concentrations reached their peak level at 24 hr (Group A: 8.4 ± 3.4 ng/mL; Group B: 7.4 ± 1.8 ng/mL) and these values were significantly higher than those in the preoperative period (Group A: 5.0 ±1.5 ng/mL; Group B: 4.8 ± 0.6 ng/mL) (P < 0.05). Forty eight hours after surgery the levels of ghrelin returned to their preoperative status. Patients’ gender, age, ASA score and type of surgical procedure did not influence the serum ghrelin levels.

**Conclusions:**

Serum ghrelin concentration appears to elevate in response to surgical stress. Future studies are needed to improve comprehension of the mechanisms underlying responses of this hormone to acute surgical stress and to evaluate their possible clinical implications.

## Introduction

Ghrelin is a 28-amino acid lipopeptide-appetite hormone and an endogenous ligand for the growth hormone secretagogue receptor. It was originally isolated from rat stomach and it has been localized in endocrine X/A-like cells in the gastric mucosa [1]. The secretion of ghrelin is stimulated by fasting and its role has been linked to the regulation of gastrointestinal and cardiovascular function as well as to the modulation of energy metabolism and immune response [2, 3]. Studies in humans have shown that ghrelin levels were increased in inflammatory bowel diseases and correlated with the severity of the disease [4]. Furthermore in Wistar Albino rats, serum ghrelin levels were increased in the first 48 hr of acute edematous and necrotizing pancreatitis [5].

Surgical injury triggers the release of stress hormones which stimulate a cascade of metabolic and endocrinologic changes, which are proportional to the severity of the operation [6]. An altered stress response has marked effects on the postoperative course of the patient. In view of this notion, many studies have investigated different aspects of the surgical stress response, such as cytokine production and mechanism of lymphocyte dysfunction [7, 8]. Recently, some researchers, influenced by the multifactorial role of ghrelin, examined the effects of surgery to the secretion of ghrelin, nonetheless to date data remain conflicting [9-12]. In this study we evaluated the changes in ghrelin serum levels in patients undergoing elective gastrointestinal surgery for either cholelithiasis or colorectal malignancies and review the current literature.

## Patients and Methods

After obtaining approval from the Ethical Committee of our hospital, all patients scheduled for abdominal surgery from January 2002 until January 2005 were evaluated for study enrollment. Inclusion criteria were age above 18 years and American Society of Anesthesiologists (ASA) physical status I or II. Exclusion criteria included the presence of hepatic, renal, metabolic or endocrine disease and severely altered nutritional status. Patients in need of blood transfusion, or corticosteroids during the perioperetive period were also excluded.

Overall 25 patients were included in the study from which informed consent was obtained. Patients were prospectively allocated in two groups based on the type of surgical operation. Group A (n = 10) patients were scheduled to undergo cholecystectomy for symptomatic cholelithiasis, whereas Group B (n = 15) patients underwent colectomy for colorectal neoplasms.

All patients received premedication with 0.02 mg/kg lorazepam 30 - 45 min before surgery. Induction of general anesthesia included intravenous infusion of remifentanil 1 µg/kg, propofol 2 mg/kg and cisatracurioum 0.2 mg/kg. Anesthesia was maintained with a mixture of sevoflurane 2-3%, air 50% and oxygen 50%. Crystalloid fluid was infused during the surgical procedure and throughout the postoperative period until 48hr after surgery, when the last blood sample was taken.

Serum ghrelin concentrations were evaluated in each patient preoperatively (T1), after the induction of general anesthesia and tracheal intubation (T2), one (T3) and five (T4) hours after the beginning of surgery and the morning of the first (T5) and second (T6) postoperative day. Each blood sample was centrifuged at 4000 g for 5 min and serum was removed and stored at -80 °C until the time of the assay. The levels of ghrelin were measured with enzyme immunoassay method (Peninsula Laboratories, Inc.USA).

A standard statistical software package SPSS (SPSS Inc, Chicago IL) was used in the analysis. Descriptive statistics were calculated for all variables. Categorical variables were analyzed with the chi-square test or Fisher's exact test as appropriate. The one-sample Kolmogorov-Smirnov test was used to test if a variable was normally distributed. All data were normally distributed and they are presented as mean ± SD. The repeated measurements of ghrelin levels between the two groups were compared with an ANOVA for repeated measures, while differences at each specific time point were evaluated with the T-test. P values less than 0.05 were considered statistically significant.

## Results

**Figure 1:**
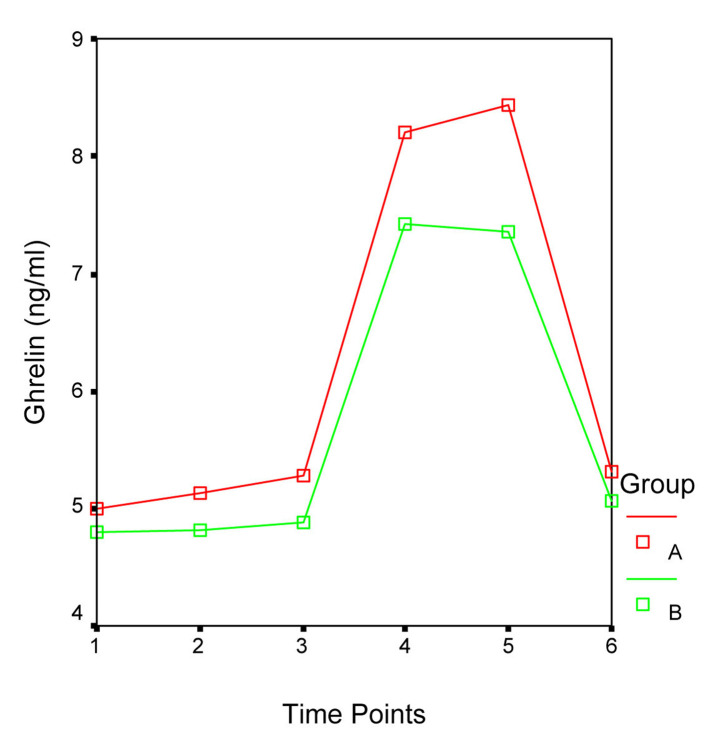
Fluctuations in the ghrelin levels during the different time points of the study. F = 0.954, P = 0.339.

Patients’ demographic data and ghrelin concentrations at each time point are presented in Table 1. In both groups serum ghrelin levels started to increase immediately after surgery, reached their peak level at 24 hr (Group A: 8.4 ± 3.4 ng/mL; Group B: 7.4 ± 1.8 ng/mL) and returned to the preoperative values at 48 hr. Statistical analysis did not reveal significant differences between Group A and B ghrelin levels during the different time point of the study. Furthermore, repeated measurements ANOVA revealed that the fluctuations in the ghrelin concentration during the study time period did not differ significantly between the two groups (F = 0.954, P = 0.339; Fig. 1). Nonetheless in both groups the ghrelin levels five (T4) hours after the beginning of surgery and in the morning of the first (T5) postoperative day were significantly higher than those preoperatively (T1), after the induction of general anesthesia and tracheal intubation (T2) and one (T3) hour after the beginning of surgery, while no differences were detected between the latter and ghrelin levels on the second (T6) postoperative day (Table 2 and 3). Patients’ gender, age and ASA score did not influence the serum ghrelin levels.

**Table 1:** Patients Demographic Data and Ghrelin Levels at Each Time Point

Type of Surgery	Cholocystectomy (n = 10)	Colectomy (n = 15)	P value
Age (mean ± SD)	46.8 ± 15.76	56.86 ± 14.32	NS
BMI (mean ± SD)	27.6 ± 4.42	27.38 ± 4.55	NS
Gender (M/F)	3/7	9/6	NS
Ghrelin 1 (ng/mL, mean ± SD)	5.0 ± 1.5	4.8 ± 0.6	NS
Ghrelin 2 (ng/mL, mean ± SD)	5.1 ± 1.5	4.8 ± 0.6	NS
Ghrelin 3 (ng/mL, mean ± SD)	5.3 ± 1.3	4.9 ± 0.5	NS
Ghrelin 4 (ng/mL, mean ± SD)	8.2 ± 2.8	7.4 ± 1.4	NS
Ghrelin 5 (ng/mL, mean ± SD)	8.4 ± 3.4	7.4 ± 1.8	NS
Ghrelin 6 (ng/mL, mean ± SD)	5.3 ± 1.3	5.0 ± 0.6	NS

Ghrelin 1: preoperatively; Ghrelin 2: After intubation; Ghrelin 3: 1 hr postoperatively; Ghrelin 4: 5 hr postoperatively; Ghrelin 5: 24 hr postoperatively; Ghrelin 6: 48 hr postoperatively; BMI: Body Mass Index.

**Table 2:** Comparison of Ghrelin Levels Between Each Time Point in Group

Time Point	(ng/mL, mean ± SD)	1	2	3	4	5	6
1	5.0 ± 1.5	N.S	N.S	N.S	0.001	0.008	N.S
2	5.1 ± 1.5	----	N.S	N.S	0.001	0.010	N.S
3	5.3 ± 1.3	----	----	N.S	0.003	0.019	N.S
4	8.2 ± 2.8	----	----	----	N.S	N.S	0.005
5	8.4 ± 3.4	----	----	----	----	N.S	0.026
6	5.3 ± 1.3	----	----	----	----	---	N.S

T 1: A preoperatively, T 2: After intubation, T 3: 1 hr postoperatively, T 4: 5 hr postoperatively, T 5: 24 hr postoperatively, T 6: 48 hr postoperatively.

**Table 3:** Comparison of Ghrelin Levels Between Each Time Point in Group B

Time Point	(ng/mL, mean ± SD)	1	2	3	4	5	6
1	4.8 ± 0.6	N.S	N.S	N.S	0.001	0.001	N.S
2	4.8 ± 0.6	----	N.S	N.S	0.000	0.000	N.S
3	4.9 ± 0.5	----	----	N.S	0.000	0.001	N.S
4	7.4 ± 1.4	----	----	----	N.S	N.S	0.000
5	7.4 ± 1.8	----	----	----	----	N.S	0.001
6	5.0 ± 0.6	----	----	----	----	---	N.S

T 1: preoperatively, T 2: After intubation, T 3: 1 hr postoperatively, T 4: 5 hr postoperatively, T 5: 24 hr postoperatively, T 6: 48 hr postoperatively.

## Discussion

This study evaluated the effect of stress during elective abdominal surgery on serum ghrelin levels. We found that the concentration of ghrelin increased significantly immediately after surgery and returned to the preoperative values in the second postoperative day. Ghrelin is a peptide mainly secreted from the stomach and its production is stimulated by the absence and attenuated by the presence of food. Growing evidence suggests that this hormone can influence the immune system in various ways [13]. According to previous animal and human studies, ghelin has been shown to enhance thymopoiesis and T cell development, to inhibit the expression of proinflammatory cytokines and may even have anti-apoptotic effects [14-16].

Each surgical operation is followed by a series of inflammatory and metabolic change that constitute the stress response. Considering the information regarding the immune/metabolic regulatory role of ghrelin some researchers have studied the pattern of this hormone secretion during surgery, with conflicting results. According to Chiesa et al, there was a significant decrease in ghrelin concentrations from baseline during the intraoperative period, in patients undergoing elective cholecystectomy [10]. On the other hand, Cetinkaya et al failed to show any differences in ghrelin levels in patients undergoing laparoscopic cholecystectomy, but did show an increase in the hormone levels in patients undergoing appendectomy [12]. Furthermore a recent study by Maruna et al revealed a significant elevation of plasma ghrelin levels 24 hours after resection of coli and subsequent return to initial status 36-48 hours after surgery [9]. In this study we showed that ghrelin increased postoperatively in both cholecystectomy and colectomy patients. Based on our, as well as on other previous results, it appears that ghrelin is an important factor in the inflammatory response following surgery, but it remains unclear how these changes in this acute phase reaction affect ghrelin concentrations. Furthermore, after taking under consideration the anti-inflammatory effect of this hormone, induction of ghrelin during the surgical stress response could possible contribute to wound healing, and postoperative infection risk but this role cannot be confirmed in our study-due to the lack of major postoperative complications in our patients-nor has been confirmed by any other clinical study.

The human response to surgical stress is related with the extent of the tissue injury; thus the levels of ghrelin should be influenced by the type of the surgical procedure. Nevertheless we failed to show such an association. Similarly Chiesa et al failed to show any differences between patients undergoing elective cholecystectomy via laparoscopy or laparotomy [10]. On the other hand Maruna et al revealed that patients undergoing colectomy had higher postoperative values of ghrelin compared to patients treated with laparotomic cholecystectomy [9]. These discrepancies could be attributed to the low statistical power of the involved studies- including ours- to the altered nutritional status of the involved patients, or to differences in the methodology, such as the timing of blood sampling.

Our study included patients with colorectal malignancies; their ghrelin levels appeared to be lower than those with cholelithiasis, but this difference was not statistically significant. To date controversy exists regarding the role of ghrelin in cancer. Ghrelin was found to stimulate cell proliferation in prostate and hepatoma cancer cell lines, implying a potential tumor-promoting role [17, 18]. On the other hand studies on breast cancer cells have documented an antiproliferative effect of ghrelin [19]. Future studies are warranted to establish the role of ghrelin, in cancer progression.

In summary, ghrelin concentrations appear to elevate in response to surgical stress. The pathophysiologic pathways that promote these changes are not fully understood. Future studies are needed to investigate the relationship between ghrelin and the acute phase response mediators. Moreover it would be quite interesting to evaluate the role of ghrelin in the development of postoperative complications as well as in the postoperative recovery of the patients’ physical and nutritional status.
